# Exploring the current molecular landscape and management of multiple myeloma patients with the t(11;14) translocation

**DOI:** 10.3389/fonc.2022.934008

**Published:** 2022-08-02

**Authors:** Michael D. Diamantidis, Sofia Papadaki, Evdoxia Hatjiharissi

**Affiliations:** ^1^ Thalassemia and Sickle Cell Disease Unit, Department of Hematology, General Hospital of Larissa, Larissa, Greece; ^2^ Division of Hematology, First Department of Internal Medicine, AHEPA General Hospital, Aristotle University of Thessaloniki, Thessaloniki, Greece

**Keywords:** Keywords: translocation t(11;14), multiple myeloma, precision medicine, BCL-2, venetoclax, genetic abnormalities, prognosis in myeloma

## Abstract

Multiple myeloma (MM) is a genetically complex disease. The key myeloma-initiating genetic events are hyperdiploidy and translocations involving the immunoglobulin heavy chain (IgH) enhancer on chromosome 14, which leads to the activation of oncogenes (e.g., CCND1, CCND3, MAF, and MMSET). The t(11;14) translocation is the most common in MM (15%–20%) and results in cyclin D1 (CCND1) upregulation, which leads to kinase activation and tumor cell proliferation. Notably, t(11;14) occurs at a higher rate in patients with plasma cell leukemia (40%) and light chain amyloidosis (50%). Patients with myeloma who harbor the t(11;14) translocation have high levels of the anti-apoptotic protein B-cell lymphoma 2 (BCL2). Multiple studies demonstrated that the presence of t(11;14) was predictive of BCL2 dependency, suggesting that BCL2 could be a target in this subtype of myeloma. Venetoclax, an oral BCL2 inhibitor, has shown remarkable activity in treating relapsed/refractory MM patients with t(11;14) and BCL2 overexpression, either as monotherapy or in combination with other anti-myeloma agents. In this review, we describe the molecular defects associated with the t(11;14), bring into question the standard cytogenetic risk of myeloma patients harboring t(11;14), summarize current efficacy and safety data of targeted venetoclax-based therapies, and discuss the future of individualized or precision medicine for this unique myeloma subgroup, which will guide optimal treatment.

## Introduction

Multiple myeloma (MM), a hematological malignancy of plasma cells (PCs), has a high level of molecular and clinical complexity (1, 2). Although MM remains an incurable disease, never before has existed such translational research progress and optimism about therapeutic options. Advances in genomic studies have increased our understanding of MM pathogenesis, demonstrating that it is not a single disease. Despite their morphological similarities, at least six different diseases, collectively known as MM, all derived from PCs, have been genetically defined (3, 4).

Early cytogenetic studies defined the most common MM abnormalities, including hyperdiploidy with chromosome (chr.) counts of 53–55 and the translocation t(11:14) (q13;q32), both described in the same paper (5). Subsequent research using interphase fluorescence *in-situ* hybridization (i-FISH) assays revealed that recurrent translocations involving the immunoglobulin heavy chain (IGH) enhancer of chr. 14 (known as 14q32 translocations) were as common in MM as hyperdiploidy (6). Both hyperdiploidy and IGH translocations are considered the main initiating events in the pathogenesis of myeloma (2, 4, 7–9).

The five most common 14q32 translocations (60% of all patients) are t(11;14), t(4;14), t(14;16), t(14;20), and t(6;14) (3, 10, 11). Translocations t(4;14), t(14;16), and t(14;20) are linked to poor prognosis and defined as high-risk cytogenetic prognostic markers, whereas patients with t(11;14), t(6;14), and/or hyperdiploidy are considered to have standard-risk disease (3, 10). The molecular classification into these subgroups has been widely used not only to predict survival but also to identify targeted therapies (9, 10, 12, 13).

MM is an incurable PC neoplasm with substantial inter- and intrapatient genetic heterogeneity, which cannot be fully captured by routine diagnostics, even within the same patient. Typically, precision medicine links molecular–genetic aberrations with matched targeted therapies. Nevertheless, the number of druggable targets in myeloma is extremely low. However, MM with t(11;14), which is susceptible to B-cell lymphoma 2 (BCL2) inhibition in the clinical setting, is the only subtype with a therapeutically exploitable vulnerability (14, 15). In addition, there is no doubt that MM with t(11;14) is a unique clinical and biological entity, corresponding most likely to B-cell myeloma (16, 17).

This review aims to characterize the molecular abnormalities associated with the t(11;14), define the cytogenetic risk of myeloma patients with t(11;14), and highlight current efficacy and safety data for venetoclax-based therapies. Furthermore, the potential of precision therapeutic intervention in this myeloma subgroup will be discussed.

## Translocation t(11:14) in MM: update on molecular and clinical finding

The t(11:14) is historically the first translocation of the 14q32 chromosome, discovered using conventional karyotype (5). Better characterization was achieved by precisely mapping the translocation breakpoints on both chromosomes (18). Following that, several researchers investigated the clinical features and outcomes of t(11:14) patients (5, 19). Numerous studies established that t(11:14) is a unique myeloma subtype, initially considered as positive prognostic translocation (20–23). However, in the novel therapies era, large studies established that patients with t(11:14) may not exhibit superior clinical outcomes compared to patients without t(11:14) or other cytogenetically standard-risk individuals (15, 24–26).

However, importantly, nearly one-third of patients with t(11;14) harbor additional high-risk cytogenetic abnormalities (27). In an early study, among 212 newly diagnosed patients, the most frequently coexisting aberrations with t(11;14) were del13q (37%), del(IGH) (33%), gain1q (20.7%), del16q (14.9%), del17p (7.7%), del1p (3.4%), and multiple gains1q (2.9%) (24). Thirty-five percent of these patients had at least two additional chromosomal abnormalities (24). The translocation t(11;14) has also been associated with cyclin D1 (CCND1) and IRF4 mutations (4), while an atypical form of t(11;14), characterized by unique FISH patterns, adding a particular biologic interest behind this lesion, has recently been reported (28).

The t(11;14) is the most frequent 14q32 translocation found in MM (15%–20%). It causes CCND1 upregulation, leading to kinase activation and tumor cell proliferation (16). A greater incidence (68%) of this translocation among relatively younger patients (<50 years) with the worse outcome has been reported (29). Furthermore, t(11;14) has been discovered in 10%–13% of individuals with monoclonal gammopathy of undetermined significance, implying that it is an early event in the disease pathogenesis (30, 31). This translocation can be found in other plasma cell-related neoplasms. It is significantly more common (~40%) in patients with plasma cell leukemia (32–34) and encountered nearly in half of those with light-chain amyloidosis (35).

African Americans or individuals with a high degree of African ancestry have an increased prevalence of the t(11;14) translocation (36, 37). This finding may partially explain the influence of race on prognosis and outcomes in MM, a complex subject with conflicting results under investigation (38, 39).

Patients with this specific translocation have a unique myeloma type and are less likely to be hyperdiploid (17). They show a two-fold increased incidence of light-chain or non-secretory myeloma, are more likely to present with bone disease, and harbor uncommon heavy-chain isotypes such as immunoglobulin M (IgM) or IgE (10, 39–42). Malignant PCs are less mature with scant cytoplasm, have a lymphoplasmacytic phenotype with CD20 expression in roughly half of the cases and, most critically, are prone to apoptosis through the anti-apoptotic protein BCL2, which is the target for venetoclax (39, 40, 43).

Because MM patients with t(11;14) overexpress BCL2, venetoclax, a highly selective oral BCL2 inhibitor, appears to be an effective therapeutic strategy (44–46). We focus on t(11:14) because it has emerged as the first predictor of response to BCL2 inhibition in MM, thereby establishing a very intriguing field of potential treatments.

## The prognostic and predictive role of t(11;14) in MM

The presence of high-risk cytogenetic features identified originally as [del(17p), t(4;14), and t(14;16)] has been extensively documented as a negative prognostic factor in MM. These patients typically have a worse outcome than standard-risk patients with t(11;14), t(6;14), and/or hyperdiploidy (42, 47, 48).

The risk classification of the t(11;14) has been a long-running discussion, most likely in the wrong direction. The t(11;14) was associated with a relatively favorable outcome and classified as a standard-risk translocation when alkylators like melphalan or cyclophosphamide were the backbone of myeloma care (49). However, recent studies showed an inferior depth of response rates, progression-free survival (PFS), and overall survival (OS) for t(11:14) patients treated with novel agents compared to standard-risk myeloma (16, 50–52). These findings question the classification of t(11;14) patients in the standard-risk subgroup, claiming that they should be classified as intermediate risk and treated appropriately (24, 25).

One of the largest studies conducted by the Mayo Clinic first showed that MM patients with t(11;14) had better PFS, time to next treatment (TNT), and OS than the non-t(11;14) translocation group (high-risk abnormalities), but worse markers than the standard-risk group, which includes trisomies or normal cytogenetics. The outcome difference remained even after excluding patients with del(17p) from all subgroups. As a result, patients with t(11;14) were reclassified as “intermediate risk” (16).

Another retrospective, single-center study including 1,000 newly diagnosed MM patients analyzed the outcome of the largest cohort of t(11:14) patients ever evaluated. All patients were treated identically with a triplet regimen (VRd) consisting of a proteasome inhibitor (bortezomib, V), an immunomodulatory drug (lenalidomide, R), and dexamethasone (d) representing the standard of care in the era of novel therapies. In this study, the presence of t(11;14) was linked to lower response rates [very good partial response (VGPR) 50% *vs*. 76%; *p* < 0.001] and subsequently to shorter median PFS (51 *vs*. 75 months; *p* < 0.001) compared to patients with standard risk (51, 52).

According to several studies, autologous stem cell transplantation (ASCT) may abrogate the unfavorable outcome of patients harboring the t(11:14) with a superior survival for transplanted African Americans compared to Whites in the United States (53). However, the ASCT impact has not been adequately explored in these patients because they are often studied together with hyperdiploid patients as having standard-risk disease (54, 55).

Moreover, the presence of multiple cytogenetic abnormalities has a greater impact on the prognosis of MM than a single high-risk aberration (47, 56, 57). Individuals with multiple gains(1q), del(1p), del(IGH), and del(13q) have a significantly worse median OS (*p* < 0.05) than patients with a sole t(11;14). Worse outcome with weaker correlation has also been observed in patients with t(11;14) together with del(17p) (*p* = 0.07 > 0.05) (24). These results indicate that MM patients with t(11;14), who carry the extrachromosomal abnormalities listed above, should be recognized as having a high-risk disease, rather than a standard-risk disease, and should be considered as at least intermediate-risk patients.

In a recent review, Gao et al. conclude that outcomes of t(11;14) MM are similar to standard-risk patients when they receive novel agent induction therapy consolidated by ASCT, regardless of coexisting with single gain(1q) or not (26). Significantly worse median OS has been observed for t(11;14) patients with multiple gains(1q), compared to single gain(1q) (24).

## Translocation t(11;14) and BCL2 protein family codependency

BCL2 is a member of the regulator protein family controlling apoptosis by either initiating or ending cell death (44). BIM, BAD, PUMA, BID, BMF, HRK, NOXA, BAK, BAX, and BIK (BH3 domain-containing proteins) comprise the main pro-apoptotic BCL2 family proteins. Conversely, MCL1, BCLW, BCLXL, BFL1, and BCL2 are the main anti-apoptotic proteins. The balance of pro- and anti-apoptotic proteins affects cell fate, which varies between cells (46).

While chronic lymphocytic leukemia and follicular lymphoma are BCL2-dependent neoplasms, MM is typically MCL1 dependent. Specifically, most myeloma cell lines (80%) are dependent on the anti-apoptotic MCL1 protein or both MCL1 and BCLXL, being the most dominant pro-survival protein in MM (44, 58–61). Thus, MCL1 inhibitors exert anti-myeloma properties (44, 46, 60).

In contrast, only 20% of myelomas favor signals through the BCL2 protein, which is a target of venetoclax (12, 44, 60–62). MM with t(11;14) has distinct biology with considerably increased BCL2 and decreased MCL1 expression (44, 60). Due to high BCL2 expression, patients with t(11;14) are dependent on anti-apoptotic BCL2 proteins (46, 58). The rationale behind the response to BCL2 inhibitors is that malignant cells exhibit anti-apoptotic properties to survive. The upregulation of BCL2 is the hallmark of resistance to apoptosis for myeloma cells. Thus, malignant PCs manage to overcome pro-apoptotic alterations, caused by hypoxia, growth factor withdrawal, or adhesion loss (12, 46).

Inflammatory cytokines such as IL-6, derived from bone marrow (BM), mediate BCL2, BCLXL, and MCL1 expression in MM, forming complex network interactions between the marrow stroma and myeloma cells (63–65). IL-6 is a paracrine factor delivered by the microenvironment, primarily by myeloid cells, promoting the growth and survival of malignant PCs (66, 67). These mechanisms could account for the complex signaling interactions between t(11;14) MM and the BM microenvironment.

Hence, there is ongoing research targeting survival pathways in MM, aiming to identify which patient subgroup will benefit more from venetoclax. Although the t(11;14) group has the highest response rates, this does not rule out the possibility of other MM subgroups responding. The response also depends on the ratio of pro- and anti-apoptotic protein expression in myeloma cells with t(11;14) (12, 46). Nevertheless, there is heterogeneity regarding the level of BCL2 and MCL1 expression (46, 61). Overall, sensitivity to venetoclax is closely associated with high BCL2 and low BCLXL or MCL1 expression levels (60, 61). Interestingly, venetoclax response does not always coincide with increased BCL2 expression (44, 46, 59, 68).

## Translocation t(11;14) and precision intervention in MM

A better understanding of the t(11;14) prognostic consequences is required to improve present and future therapy options in MM. Establishing the t(11:14) translocation status is crucial for developing a personalized treatment myeloma strategy. Recently, a plethora of clinical trials using venetoclax, either alone or in combination with other drugs, has shown remarkable activity, particularly in the relapsed/refractory (R/R) patients with t(11:14) and/or BCL2 overexpression (69–73). We present an update on clinical trials targeting this specific translocation with an emphasis on response rates and toxicity (**Table 1**).

**Table 1:** Clinical trials involving venetoclax.

Reference	Trial phase	Targeted drugs/dose	Study MM population	Median lines of prior therapy/refractoriness	Response rates	Grade 3–4 toxicity	Study status	Clinical trial identifier
Kumar S et al., 2017 (74)	Phase I Interventional Non-randomized	Venetoclax [300, 600, 900, 1,200 mg/day maximum tolerated dose (NR)], 1,200 mg/day (expansion)	R/R, 66 pts 30+ t(11;14)	5 prior lines Bortezomib (70%) Lenalidomide (77%) Pomalidomide (53%) ASCT (76%)	Total: ORR: 21% VGPR or better: 15% t(11;14): ORR: 40% VGPR or better: 27% DOR: 9.7 mts	Thrombopenia (26%) Neutropenia (21%) Anemia (14%) Lymphopenia (15%) Back Pain (8%) Pneumonia (8%)	Completed	NCT01794520
Kaufman JL et al., 2020 (71)	Phase I/II, non-randomized	Venetoclax (800 mg/day) Dexamethasone (40 mg/wk)	R/R t(11;14) 20 pts (phase I) 31 pts (phase II)	3 prior lines (phase I) 5 prior lines (phase II)	Phase I: ORR: 60% VGPR or better: 30% DOR: 12.4 mts Phase II: ORR: 48% VGPR or better: 36% DOR, OS: NR	Phase I: Thrombopenia (10%) Neutropenia (10%) Anemia (12%) Lymphopenia (20%) TLS: 10% Phase II: Similar 11 deaths 8 progression 2 adverse event	Completed	NCT01794520
Costa LJ et al., 2021 (72)	Phase II, non-randomized	Venetoclax Maximum tolerated dose (NR) 800 mg/day (expansion) Carfilzomib 70 mg/m^2^ (expansion) Dexamethasone	R/R, 49 pts	1–3 prior lines	t(11;14) median 2 prior lines: ORR: 92% VGPR or better: 85%	Novel safety concerns have not arisen	Ongoing	NCT02899052
Gasparetto C et al., 2021 (75)	Phase II, non-randomized	Venetoclax (400 mg/day) Pomalidomide (4 mg/day) Dexamethasone (40 mg/wk)	R/R, 8 pts		t(11;14) Positive or negative status	Toxicity	Terminated	NCT03567616
Mateos MV et al., 2020 (76)	Phase III, randomized, CANOVA	Venetoclax (400 mg/day) Dexamethasone (40 mg/wk) vs. Pomalidomide (4 mg/day) Dexamethasone **(**40 mg/wk)	244 pts (estimated)	≥2 prior lines Lenalidomide refractory (100%)			Ongoing	NCT03539744
Moreau P et al., 2017 (69)	Phase 1b, non-randomized	Venetoclax (100, 200, 300, 400, 500, 600, 800, 1,000, 1,200 mg/day) Bortezomib (1.3 mg/m^2^) Dexamethasone (20 mg)	R/R, 66 pts t(11;14): 14%	3 prior lines Bortezomib (39%) Lenalidomide (53%) ASCT (59%)	Total: ORR: 67% VGPR or better: 42% DOR: 9.7 mts RR: 89% (1–3 prior lines), 50% (4–6), 11% (>6)	Thrombopenia (29%) Neutropenia (14%) Anemia (15%) Pneumonia (8%)	Completed	NCT01794507
Kumar S et al., 2020 (70) Kumar S et al., 2021 (77)	BELLINI, phase III, randomized, double blind Final cutoff data: March 2021 Median follow-up: 45.6 mts	Venetoclax (800 mg/day) Bortezomib (1.3 mg/m^2^) Dexamethasone (20 mg) vs. Bortezomib (1.3 mg/m^2^) Dexamethasone (20 mg)	R/R VEN: 194 pts t(11;14): 10% Placebo: 97 pts t(11;14): 15%	1–3 prior lines	Median OS: NR in both arms PFS median (total): VEN (23.4 mts) vs. placebo (11.4 mts) PFS median t(11;14): VEN (36.8 mts) vs. placebo (9.3 mts) ORR: VEN 82% vs. placebo 68% VGPR or better: VEN 59% vs. placebo 36%	Total deaths: VEN 78 (40%) vs. placebo 36 (37%) Serious infections: Ven: 35% placebo: 29% Fatal infections: VEN 9 pts vs. placebo 0 pts Thrombopenia Neutropenia Pneumonia Anemia Diarrhea	Suspended, due to safety	NCT02755597
Kaufman JL et al., 2020 (71)	Phases I and II, randomized Preliminary results (May 2020)	Venetoclax Various Doses Daratumumab 1800mg SC Or 16mg/kg IV Bortezomib (1.3mg/m^2^) Dexamethasone 20 mg, cycles 1–3: 20 or 40 mg/wk, cycles 4–8: 20 mg/mth c. 9+	Part I (VEN-DARA-DEXA): t(11;14): 24 pts Part II (VEN-DARA-BORT-DEXA): 24 pts t(11;14): 6		ORR: >90%	Similar to previous VEN trials Infections: 9 pts Additional non-VEN arm (DARA-BORT-DEXA) to compare VEN toxicity	Ongoing	NCT03314181
Maples KT et al., 2020	Retrospective	Venetoclax	t(11;14) 68 pts		Total: ORR: 71% VGPR or better: 48.5% Median PFS: 14.1 or 23.2 mts for <3 prior lines		Completed	

NR, not reached; DOR, duration of response; OS, overall survival; PFS, progression-free survival; ORR, overall response rate; RR, response rate; VGPR, very good partial response; TLS, tumor lysis syndrome; VEN, venetoclax; DARA, daratumumab; DEXA, dexamethasone; BORT, bortezomib; mts, months; pts, patients; MM, multiple myeloma.

The first evidence of the substantial clinical activity of venetoclax as a single agent was reported in the R/R myeloma setting in phase 1 clinical trials. Patients with very advanced MM were enrolled with a median of five prior therapies, the majority being refractory to both bortezomib and lenalidomide. Apart from two patients, all the rest of the responders (40%) were t(11;14) positive (74). Nevertheless, responses were also observed in non-t(11;14) patients who received venetoclax, although to a lesser extent (74).

Additionally, the efficacy of venetoclax was confirmed in R/R MM, when added to bortezomib and dexamethasone. This combination demonstrated 90% overall response rate (ORR) and >64% VGPR in individuals not refractory to bortezomib (69). Subsequently, in the phase III BELLINI trial (NCT02755597), venetoclax (800 mg/day) in combination with bortezomib and dexamethasone (Ven-Vd) demonstrated superior efficacy to placebo plus Vd in patients with t(11;14) R/R MM. After a median 4-year follow-up, the venetoclax arm achieved a PFS of 36.8 months compared to 9.3 months in the placebo arm [hazard ratio (HR), 0.12; 95% CI, 0.03–0.44; *p* = 0.0014]. The greatest benefit was observed for those who had high BCL2 expression or who were t(11;14) positive. In neither arm was the median OS reached (HR, 0.61; 95% CI, 0.16–2.32; *p* = 0.4654) (70, 77). In addition, while the trial’s original analysis revealed a mortality increase among venetoclax-treated patients, the updated analysis did not report any further increase in early deaths in the venetoclax arm. The BELLINI study was halted due to the abovementioned toxicity and the increased mortality rate in the venetoclax arm (40%, 78 deaths). Most deaths were due to infectious complications, especially during disease progression. Moreover, they were mainly confined to patients with low BCL2 expression of the non-t(11;14) subgroup (70, 77).

Venetoclax has been examined in combination with carfilzomib and dexamethasone (NCT02899052) in a non-randomized, phase 2, open-label trial (72) as well as with daratumumab and dexamethasone with or without bortezomib (NCT03314181, a randomized phase 1 study) in cytogenetically unselected patients with R/R MM (73). The latter study demonstrated outstanding clinical responses with no new safety signals, confirming again that venetoclax is more effective in t(11;14) patients, with a high and deep ORR over 90% (73).

Based on the BELLINI trial results, the clinical development of venetoclax is currently limited to t(11;14) patients. For example, the CANOVA trial (NCT03539744) is a phase 3, randomized trial comparing venetoclax plus dexamethasone *vs*. pomalidomide plus dexamethasone in t(11:14) R/R MM patients (76). Nevertheless, a phase 2, non-randomized study of venetoclax in combination with pomalidomide and dexamethasone in R/R MM (NCT03567616), including patients with positive or negative t(11:14) status, was terminated due to limited enrollment and toxicity (75).

Preliminary data outside of clinical studies also confirmed that venetoclax-based combinations achieve remarkable responses in heavily pretreated R/R MM (78–80). While the BELLINI trial showed that a proper patient selection may reduce the risk of venetoclax-associated side effects, the natural history of myeloma relapsing on venetoclax is largely unknown. The first retrospective study to assess real-world outcomes from the time of venetoclax refractoriness demonstrated a median OS of 31.4 months. These findings support the use of venetoclax early in the treatment of the t(11;14) patient group, challenging the notion that venetoclax resistance results in a more aggressive disease phenotype (81).

These data significantly support BCL2-directed therapy, such as venetoclax in t(11;14) patients, comprising the first step toward a precise treatment strategy in myeloma, namely, “precision medicine” (9, 14, 61, 78, 82). We should underline, however, that venetoclax is still investigational and should only be used in the context of clinical trials. Although applying venetoclax outside of a clinical trial is sometimes helpful, we are still learning about the drug.

Currently, venetoclax is administered in the R/R setting to patients with MM harboring t(11;14). However, we assume that the drug could and should be a first-line treatment option. To the best of our knowledge, there are no ongoing clinical trials or research studies in this use. Patients with t(11;14) MM have an unmet need for well-designed clinical trials evaluating the safety and efficacy of venetoclax as first-line therapy.

## Response and resistance to venetoclax

Venetoclax is not beneficial for all patients with t(11;14). Certain patients may have a remarkable venetoclax response, while others do not. Although the biology underlying this heterogeneity is unknown, both *de-novo* and acquired resistance can occur (81). BCL2 expression has been correlated with a significantly higher response to venetoclax. Conversely, the expression of MCL1 and BCLXL in cell lines has been linked to a poor response (58, 60, 82). However, ongoing studies using BH3 profiling, a novel *ex-vivo* functional technique for determining a cancer cell’s reliance on anti-apoptotic proteins, revealed that MM is a diverse disease in terms of anti-apoptotic protein dependency and cannot be considered exclusively BCL2, BCLXL, or MCL1 dependent (59, 60, 83).

Contrary to prior findings, Gupta et al. demonstrated that t(11;14) and CCND1 may not directly affect the likelihood of response to venetoclax in MM (68). They specifically identified other factors contributing to the venetoclax response, including increased expression of a signature panel of B-cell genes, exclusively observed in venetoclax-sensitive t(11;14) patients. Additionally, the authors developed a predictive flow cytometric score for venetoclax response including B markers (CD20, CD79A) used to detect venetoclax-sensitive myeloma without relying on t(11;14) (68).

Intriguingly, a patient subset lacking t(11;14) responds to venetoclax. This was observed in a subset of high-risk patients harboring the t(14;16) translocation, demonstrating a remarkable response to venetoclax, most likely due to their elevated CD2 expression signature (MS4A1 or CD20, CD79A, VPREB3, PIK3IP1), despite the absence of t(11;14) (68). They also reported that BIM binding to BCL2 is linked with venetoclax response, a finding supported by decreased sensitivity to venetoclax in BIM knockout mice.

Another study demonstrated that an immature plasma cell phenotype in myeloma patients with t(11;14) consisting of significantly low CD38 and CD138 expression and high levels of B-cell markers such as CD79A and PAX5, along with an increased BCL2/BCL2L1 ratio, was highly susceptible to venetoclax treatment and less sensitive to daratumumab-based therapies, supporting further the hypothesis that venetoclax sensitivity is predicted by a robust B-cell myeloma phenotype (84). Interestingly, CYLD inactivation and 1q gains were also reported to predict venetoclax response (85). In contrast, high neuregulin-2 expression has been linked to venetoclax resistance in non-t(11;14) MM (86). The shift in myeloma cell dependence from BCL2 to MCL1 or BCLXL is the hallmark of acquired resistance to venetoclax in t(11;14) MM, as most responders eventually relapse (87, 88). Another resistance mechanism has recently been identified as a *de-novo* D111A mutation in BCL2 caused by venetoclax treatment (88).

## Concluding remarks

MM is a genetically complex disease associated with a number of recurrent translocations and mutations influencing prognosis, clinical presentation, and treatment response. Regardless of advances in the molecular disease landscape, this progress has not been translated into clinical benefit, primarily because the majority of drugs still target the neoplastic tumor burden rather than specific mutations, rearrangements, or underlying genomic defects of PCs (89). A remarkable exception seems to be venetoclax, the first targeted medication with clinical efficacy proven in a patient subset carrying a certain cytogenetic profile, like t(11;14). Defining MM patients who are most likely to benefit from venetoclax treatment is still a work in progress. Detection of features associated with venetoclax response is crucial. A recent study showed that a specific B-cell gene signature predicts better and stronger responses than the t(11;14) translocation (68).

Myeloma with t(11;14) has distinct biology with considerably increased expression of the anti-apoptotic protein BCL2. The translocation t(11;14) has been associated with a more severe disease phenotype in patients who carry additional chromosomal or molecular abnormalities and this should be further evaluated. A better understanding of the prognostic consequences of the t(11;14) translocation is necessary to guide existing and future therapy options (46, 68).

Despite the fact that our understanding from t(11:14) has been translated into clinical practice, the benefits are applicable to some patients. Most patients do not respond for long and their disease worsens. The combination of venetoclax or BCL2 inhibitors with other anti-myeloma drugs or novel intervention strategies has the potential to improve clinical outcomes.

## Author contributions

D.D.M: collected and assembled the data, generated the main body and tables of the manuscript S.P. generated sections of the manuscript E.H: conceptualized and designed the document, guided the co-authors, generated sections of the manuscript, and was responsible for the final writing of the document. The entire manuscript was reviewed and approved by all co-authors.

## Conflict of interest

The authors declare that the research was conducted in the absence of any commercial or financial relationships that could be construed as a potential conflict of interest.

## Publisher’s note

All claims expressed in this article are solely those of the authors and do not necessarily represent those of their affiliated organizations, or those of the publisher, the editors and the reviewers. Any product that may be evaluated in this article, or claim that may be made by its manufacturer, is not guaranteed or endorsed by the publisher.

## Abbreviations

ASCT, autologous stem cell transplantation; BAD, BCL2-associated death; BAK, BCL2 antagonist/killer; BAX, BCL2-associated X protein; BCL2, B-cell lymphoma 2; BCL2L1, B-cell lymphoma 2-like protein 1; BCLXL, B-cell lymphoma-extra large; BH3, BCL2 homology domain 3; BID, BCL2-interacting domain; BIK, BCL2-interacting killer; BIM, BCL2-interacting mediator; BM, bone marrow; BMF, B-cell maturation factor; CCND1, cyclin D1; chr., chromosome; CYLD, cylindromatosis tumor suppressor protein; HR, hazard ratio; i-FISH, interphase fluorescence *in-situ* hybridization; IgE, immunoglobulin E; IGH, immunoglobulin heavy chain; IgM, immunoglobulin M; IL-6, interleukin-6; IRF4, interferon regulatory factor 4; MCL1, myeloid cell leukemia 1; mg, milligrams; MM, multiple myeloma; ORR, overall response rate; OS, overall survival; PAX5, paired box protein 5; PCs, plasma cells; PFS, progression-free survival; PIK3IP1, PI3K-interacting protein 1; PUMA, p53-upregulated modulator of apoptosis; R/R, relapsed/refractory; TNT, time to next treatment; Ven-Vd, venetoclax–bortezomib–dexamethasone; VGPR, very good partial response; VRd, bortezomib-V, lenalidomide-R, dexamethasone-d.
